# Zinc Methionine Improves the Growth Performance of Meat Ducks by Enhancing the Antioxidant Capacity and Intestinal Barrier Function

**DOI:** 10.3389/fvets.2022.774160

**Published:** 2022-01-31

**Authors:** Yaqi Chang, Huangyao Tang, Zhenyu Zhang, Ting Yang, Bing Wu, Hua Zhao, Guangmang Liu, Xiaoling Chen, Gang Tian, Jingyi Cai, Fali Wu, Gang Jia

**Affiliations:** ^1^Key Laboratory of Animal Disease-Resistance Nutrition, Ministry of Education, Ministry of Agriculture and Rural Affairs, Institute of Animal Nutrition, Sichuan Agricultural University, Chengdu, China; ^2^Institute of Animal Husbandry and Veterinary Medicine, Meishan Vocational Technical College, Meishan, China; ^3^Chelota Group, Guanghan, China

**Keywords:** zinc methionine (Zn-Met), antioxidant capacity, intestinal barrier, growth performance, meat duck

## Abstract

This study was conducted to investigate the effects of zinc methionine (Zn-Met) on the growth performance, antioxidant capacity and intestinal barrier function of meat ducks. Three hundred and sixty 1-day-old male Cherry Valley ducks were randomly divided into 6 groups with 6 replicates (10 birds each), and fed diets with 0, 30, 60, 90, 120 or 150 mg/kg Zn for 35 d. The results indicated that dietary supplementation with Zn-Met substantially increased the average daily gain (ADG), and reduced the feed to gain ratio (F/G) during 1–35 d (*P* < 0.05). Dietary Zn-Met markedly increased the activity of superoxide dismutase (SOD), catalase (CAT) and glutathione (GSH), and reduced the malondialdehyde (MDA) content in the jejunum (*P* < 0.05). The mRNA expression levels of critical antioxidant enzymes such as *SOD, CAT*, and *nuclear factor erythroid 2-related factor 2* (*Nrf2*) were increased by Zn in the jejunum (*P* < 0.05). Supplementation with 60, 90, 120, and 150 mg/kg of Zn significantly reduced the diamine oxidase (DAO) activity in the serum (*P* < 0.05). Different levels of Zn can increase the mRNA expression of occluding (*OCLN)* and zonula occludens-1 (*ZO-1)* in the jejunum (*P* < 0.05). Diets supplemented with zinc significantly increased the content of mucin2 (MUC2), secretory immunoglobulin A (sIgA), immunoglobulin A (IgA) and immunoglobulin G (IgG) in the jejunum of meat ducks (*P* < 0.05). The 16S rRNA sequence analysis indicated that 150 mg/kg of Zn had a higher relative abundance of Verrucomicrobia and Akkermansia in cecal digesta (*P* < 0.05). In conclusion, Zn-Met improved the growth performance of meat ducks by enhancing intestinal antioxidant capacity and intestinal barrier function. This study provides data support for the application of Zn-Met in meat duck breeding.

## Introduction

With the issuing of anti-antibiotic policy, it is necessary to study the effects of antibiotic substitutes on poultry health, such as probiotics (1), organic acids (2) and trace elements. Zinc (Zn) is one of the essential trace elements to maintain the normal life activities of animals. It plays an important role in improving animal growth performance, enhancing antioxidant capacity and immunity, promoting intestinal development, and regulating intestinal flora (3, 4). But in excess, the usage of inorganic Zn has detrimental effects, such as low digestibility, and polluting the environment through feces (5), while Zn-methionine (Zn-Met) is a kind of chelate with a unique ring structure formed by inorganic Zn and methionine under specific reaction conditions (6, 7). Compared with inorganic Zn, the appropriate level of Zn-Met is more conducive to improve the growth performance and carcass yield of broilers (8), improve the bioavailability of Zn, reduce the excretion of Zn and decrease the pollution of trace elements to the environment (9, 10). Oxidative reaction is the basis of many biochemical pathways and cell functions, the imbalance of oxidation and antioxidation *in vivo* will lead to oxidative stress (11), which will produce a large number of oxidation intermediates and have a great negative impact on the growth performance and health status of poultry (12, 13). Zn exerts antioxidant function is probably through its being a co-factor of the antioxidant enzyme superoxide dismutase (SOD), thereby maintaining protein sulfydryl groups, oxidative damage of the cell membrane by free radicals occurs during Zn deficiency, thus altering the status of antioxidant enzymes, and substances (14), thus it is necessary to study the role of zinc in antioxidant enzymes. In addition, the intestinal physical barrier forms the first line of defense between the intestinal cavity and the internal environment, it is mainly composed of a single layer of columnar epithelial cells and the tight junction between cells, which not only allow the absorption of nutrients, but also prevent the invasion of pathogens, and toxins (15, 16). Studies have confirmed that some factors, such as high-density feeding environment, heat stress, and toxins, can induce excessive production of pro-inflammatory factors and reactive oxygen species, thereby destroying the intestinal barrier function (17, 18). Intestinal health is closely related to the normal growth and development of animals. Intestinal barrier function is considered to be one of the potential causes of decreased productivity and increased incidence of healthy issues in animals (19). For intensive and large-scale breeding poultry, high-density farming will lead to intestinal barrier dysfunction and decreased production performance (20), which will cause considerable economic loss to animal husbandry production. Therefore, it is still a big challenge for poultry nutritionists to improve the intestinal antioxidant capacity, and intestinal barrier function of meat ducks through nutrition strategies, maintain the intestinal health of meat ducks, and then improve the growth performance of meat ducks.

It is well known that Zn can improve the antioxidant capacity of animals (21). Nuclear factor erythroid 2-related factor 2 (*Nrf2*) is a key transcription factor regulating antioxidant defense response (22). Zhang et al. (23) reported that Zn containing attapulgite can improve the activities of catalase (CAT), SOD, and Cu/Zn superoxide dismutase (Cu/Zn-SOD) in the intestine of *Pagrosomus macrocephalus*. Further study found that Zn containing attapulgite can increase the gene expression of *Cu/Zn-SOD, Mn-SOD*, and *CAT* by activating *Nrf2* signaling pathway in the intestine of *Pagrosomus macrocephalus* (23). Zn also improved intestinal barrier function to some extent. The results showed that nano ZnO could significantly reduce the activity of diamine oxidase (DAO) in serum, decrease the intestinal permeability, increase the gene expression of tight junction protein and enhance the intestinal barrier function of weaned piglets (24). In addition, Zn containing clinoptilolite can significantly increase IgG and sIgA contents in the jejunal mucosa of broilers (25), suggesting that Zn can regulate intestinal immune function in animals. Zn deficiency can change the composition and function of intestinal flora of broilers, and reduce the richness and diversity of species (26). Yu et al. (27) found that ZnO can significantly increase the relative abundance of verruco microbia in ileal chyme of weaned piglets and regulate the intestinal microflora of weaned piglets. In conclusion, the above studies show that there is an important correlation between dietary Zn supplementation and intestinal antioxidant and barrier function. Previous studies in our laboratory have found that zinc sulfate (ZnSO_4_) can improve the intestinal barrier of meat ducks (28). However, the effect of Zn-Met on the intestinal health of meat ducks is rarely reported. Therefore, this experiment was conducted to explore the effects of Zn-Met on growth performance, intestinal antioxidant capacity, intestinal physical barrier, chemical barrier, intestinal immune function and intestinal microbial flora of meat ducks.

## Materials and Methods

### Experimental Design and Animal Management

The experimental procedures for animal trials were conducted in accordance with the Chinese guidelines for animal welfare and approved by the Animal Health and Care Committee of Sichuan Agricultural University (no.20181203).

A total of 360 one-day-old male Cherry Valley ducks (48.09 ± 0.43 g, Sichuan Mianying Breeding Duck Co. Ltd. Mianyang. China) were selected and randomly allocated to 6 dietary treatments with 6 replicates (10 birds each). The six treatments were provided with a basal diet and diets supplemented with 30, 60, 90, 120, 150 mg/kg Zn from Zn-Met, respectively. Birds were offered starter (1–14 d) and finisher (15–35 d) diets as pelleted feed. Diets are formulated in line with the (29) recommendation and the feed composition and nutrient levels are shown as in Table 1. The addition of graded Zn-Met caused the different content of methionine and we corrected it by addition of extra methionine in the premixes. Hence all nutrients were kept at the same levels expect for the zinc content. The measured values of Zn are shown in Table 2. During the trial, all ducks were raised in cages (1 × 0.75 × 0.75 m) with controllable temperature and humidity (25–32°C and 65% respectively). At 2 and 5 weeks of age, feed consumption and body weights were recorded by pen basis after 8 h of feed withdrawal, and mortality was recorded daily to correct for feed: gain data. The birds were provided with free access to feed and drinking water. The living environment of the ducks were in accordance with the animal welfare guidelines.

**Table 1:** Composition and nutrient levels of basal diets (%, air-dry basis).

**Items**	**Content**	
	**1 to 14 days**	**15 to 35 days**
**Ingredients,%**		
Corn	61.10	67.07
Soybean meal	34.72	27.40
Wheat bran	0.50	2.00
Limestone	0.78	0.75
CaHPO_4_	2.04	1.97
NaCl	0.3	0.30
Choline chloride,50%	0.15	0.15
*DL*-Met	0.15	0.13
*L*-Lys∙HCl	0.03	
Mineral premix^a^	0.20	0.20
Vitamin premix^b^	0.03	0.03
Total	100.00	100.00
**Nutrient levels^c^**		
AME/(MJ /kg)	12.14	12.15
Crude Protein	20.00	17.51
Ca	0.90	0.85
Available P	0.42	0.40
Lys	1.10	0.91
Met	0.46	0.41
Trp	0.24	0.20
Thr	0.77	0.67
Zn (mg/kg)	29.53	28.96

a *Mineral premix provided the following per kg of diets: Fe (as Ferrous Sulfate) 80 mg, Cu (as Copper Sulfate) 8 mg, Mn (as Manganese Sulfate) 70 mg, I (as Potassium Iodide) 0.40 mg, Se (as Sodium Selenite) 0.30 mg*.

b *Vitamin premix provided the following per kg of diets: VA 12 000 IU, VD_3_ 1 000 IU, VE 7.5 IU, VB_1_ 0.6 mg, VB_2_ 4.8 mg, VB_6_ 1.8 mg, VB_12_ 0.09 mg, D-Pantothenic Acid 7.5 mg, Nicotinic Acid 10.5 mg, Folic Acid 0.15 mg*.

c *Calculated from tables of feed composition and nutritive values provided by feed database in China (2018)*.

**Table 2:** Analyzed Zn concentration of diets (air-dry basis).

**Items**	**Dietary Zn levels (mg/kg)**	**Analyzed Zn (mg/kg)**	
		**1–14 d**	**15–35 d**
Zn-Met	0	27.96	32.49
	30	53.60	59.85
	60	93.66	95.76
	90	117.59	119.97
	120	148.45	155.20
	150	184.52	184.35

### Sample Collection

On the 36th day of the experiment, one duck of the average pen body weight was randomly selected from each replicate. Blood samples were collected from the jugular vein at 8:00 h after 12 h of fasting. Then, the samples were centrifuged at 3,000 g at 4°C for 15 min to obtain serum. The serum samples were stored at −20°C for subsequent analysis. After blood collection, 36 ducks were anesthetized by intravenous injection with sodium pentobarbital (30 mg/kg BW) and slaughtered. The jejunum and ileum mucosa were scraped and placed in a cryopreservation tube for the determination of intestinal antioxidant enzyme activity. Part of the jejunum and middle ileum were quickly removed for determination of immunoglobulin content and tight junction protein gene expression. Cecal chyme was taken for microbiological analysis. All samples were stored in −80°C until analysis.

### Biochemical Analysis

#### Determination of Zinc Content

Under the manufacturer's instructions, briefly, 0.5 g of feed samples were subjected to digestion with 10 mL HNO_3_ in digestion vessels for overnight digestion at room temperature, following digestion the samples were heated to 365°C until the solution became clear and colorless. Then, the samples were evaporated to near dryness and diluted to 10 mL with 0.5% of HNO_3_ before analysis. Zinc concentration in feed was determined by a ContrAA-700 high-resolution continuum source atomic absorption spectrometer equipped with flame (HR-CS FAAS, Analytik Jena, Germany) at 213.857 nm wavelength.

#### Intestinal Antioxidant Parameters

The mucosa samples of jejunum and ileum were homogenized in ice-cold saline solution (1:9, w/v), and then the mucosal supernatant was prepared after centrifugation at 2,500 g for 10 min at 4°C to determine the levels of intestinal antioxidant indexes. The intestinal mucosa antioxidant parameters including total antioxidant capacity (T-AOC), superoxide dismutase (SOD), malondialdehyde (MDA), catalase (CAT) and glutathione peroxidase (GSH-Px), glutathione (GSH) were measured by the commercial kits (Nanjing Jiancheng Institute of Bioengineering, Jiangsu, China).

#### Serum and Intestinal Biochemical Indicators

The concentration of DAO in serum and the concentrations of MUC2, sIgA, IgA, IgG, and IgM in jejunum and ileum were determined by the instructions of the commercial Enzyme-Linked Immunosorbent Assay (ELISA) kits bought from Jiangsu Meimian industrial Co., Ltd (Jiangsu, China). All procedures were guided by manuals of the kits.

#### Total RNA Extraction, Reverse Transcription and Real-Time PCR

Total RNA was isolated from frozen jejunal samples using trizol reagent (Takara Biotechnology Co., Ltd., Dalian, China) according to the instruction of the manufacturer. Briefly, about 0.1 g tissues were put into a mortar and grinded with 1 mL trizol reagent. The purity and concentration of total RNA were detected by using a spectrophotometer (NanDrop, Gene Company Limited, Guangzhou, China). The absorbance of the sample (260/280 nm) was measured between 1.8 and 2.0. For each sample, reverse transcription was performed using a PrimeScript™ RT reagent kit with cDNA Eraser (Takara Biotechnology Co., Ltd). The following conditions were used: 42°C for 2 min, then 37°C for 15 min, followed by 85°C for 5 s.

Quantitative real-time PCR was performed to analyze the expression levels of β-actin, CAT, SOD, Nrf2, OCLN, ZO-1, ZO-2, ZO-3 in the jejunum using SYBR Premix Ex Taq II (Tli RNaseH Plus) reagents (TakaRa, Dalian, China) and the CFX96 Real-Time PCR Detection System (Bio-Rad). Primers were synthesized in Jinweizhi Biotechnology Co., Ltd. The primer sequence was shown in Table 3. The reaction was performed in a volume of 10 μL consisting of 5 μL SYBR Premix Ex TaqTM, 1 μL reverse primer, 1 μL forward primer, 2 μL double distilled water and 1 μL of cDNA. The thermal cycler parameters were as follows: 95°C for 30 s, 40 cycles of 95°C for 5 s, and 60°C for 30 s, and 72°C for 5 min. The generated Gene-specific amplification products were confirmed by melting curve analysis after each real-time quantitative PCR assay. The β*-actin* was used to standardize the mRNA expression level of target genes, which calculated based on the 2^−ΔΔCt^ method (30). Each sample was repeated in triplicate.

**Table 3:** Real-time PCR primer sequences.

**Gene name**	**Accession no**.	**Primer**	**Primer sequences (5′ to 3′)**	**Size (bp)**
*Occludin*	XM_013109403.1	Forward	CAGGATGTGGCAGAGGAATACAA	160
		Reverse	CCTTGTCGTAGTCGCTCACCAT	
*ZO-1*	XM_013104936.1	Forward	ACGCTGGTGAAATCAAGGAAGAA	255
		Reverse	AGGGACATTCAACAGCGTGGC	
*ZO-2*	XM_013093747.1	Forward	ACAGTGAAAGAAGCTGGCGTAG	131
		Reverse	GCTGTATTCCCTGCTACGGTC	
*ZO-3*	XM_005019888.2	Forward	CAACATCCCTGACATGGAAGACAT	187
		Reverse	TGTGTTCGTGTTGGTTGCGG	
*Nrf2*	XM_005013555.2	Forward	TGTTGAATCATCTGCCTGTG	172
		Reverse	TAAGCTAGGTGGTCGAGTGC	
*SOD*	XM_013097859.1	Forward	GGAGGAGTAGCAGATGTGGAAA	190
		Reverse	AGCACTTGGCTATTCCGATG	
*CAT*	KU048802.1	Forward	CTGTTGAGGAAGCAGGAAGG	247
		Reverse	TCCGCAAAGTAATTGACAGG	
*β-actin*	EF667345.1	Forward	AGAAATTGTGCGTGACATCAA	227
		Reverse	GGACTCCATACCCAAGAAAGAT	

#### Sequencing of 16S rRNA

The 16S rDNA sequencing was used to analyze the composition of the microbial community in the cecal chyme. The samples to be tested were sent to Hangzhou Lianchuan Biotechnology Co., Ltd for analysis. The general experimental procedures were as follows:(1) DNA extraction and detection;(2) the PCR amplification;(3) product purification;(4) library preparation and quality control;(5) computer sequencing. Alpha diversity indices including Chao1, Simpson, and Shannon index were calculated by QIIME. Principal component analysis (PCA) was performed using R vegan package.

### Statistical Analysis

Data were analyzed using one-way ANOVA (with Duncan's multiple comparisons test) of using SPSS 21.0 for Windows (Chicago, IL, USA). Then regression analysis was employed to test the linear (L) and quadratic (Q) effects. Figures were generated by Graphpad Prism 8.0 (Graphpad Software Inc., La Jolla, CA). The data are presented as the mean ± SEM. *P*-values <0.05 were considered significant different.

## Results

### Effects of Zn-Met on Growth Performance of Meat Ducks

The effect of Zn-Met on growth performance of meat ducks are shown in Table 4. There was no significant difference in the initial body weight of ducks among all treatments (*P* > 0.05). Dietary supplementation with Zn-Met significantly improved the BW of meat ducks at 35 d (*P* < 0.05). Compared with the basal diet group, different levels of Zn significantly increased the ADG during 15–35 d and 1–35 d (*P* < 0.05), but no significant effect on the ADG from 1 to 14 d was observed (*P* > 0.05). Compared with the basal diet group, different levels of Zn significantly reduced the F/G of meat ducks during 1–35 d (*P* < 0.05), and the F/G changed quadratically with the increase of Zn during the whole period (*P* < 0.05), but dietary supplementation with Zn-Met had no significant effect on the F/G of meat ducks during 1–14 d and 15–35 d (*P* > 0.05).

**Table 4:** Effect of dietary Zn-Met on growth performance of ducks.

**Items**	**Dietary Zn levels (mg/kg)**						***P*** **-value**		
	**0**	**30**	**60**	**90**	**120**	**150**	**AVONA**	**Linear**	**Quadratic**
**BW (g)**									
1 d	48.05 ± 0.13	47.98 ± 0.12	48.13 ± 0.13	48.18 ± 0.09	48.20 ± 0.07	47.97 ± 0.07	0.479	0.753	0.414
14 d	678.93 ± 13.95	678.25 ± 12.90	657.17 ± 7.55	678.42 ± 12.46	669.92 ± 8.98	659.58 ± 11.67	0.596	0.274	0.602
35 d	2400.85 ± 15.90^b^	2547.60 ± 22.07^a^	2491.67 ± 39.78^ab^	2546.60 ± 42.66^a^	2512.50 ± 23.55^a^	2520.65 ± 45.15^a^	0.042	0.271	0.269
**ADG (g)**									
1–14 d	45.06 ± 0.99	45.02 ± 0.92	43.50 ± 0.54	45.02 ± 0.89	44.41 ± 0.64	43.69 ± 0.84	0.594	0.268	0.595
15–35 d	86.10 ± 1.26^b^	93.47 ± 1.37^a^	91.73 ± 1.82^a^	93.42 ± 1.80^a^	92.13 ± 1.03^a^	93.05 ± 2.27^a^	0.029	0.192	0.198
1–35 d	70.60 ± 0.47^b^	74.92 ± 0.65^a^	73.27 ± 1.17^ab^	74.89 ± 1.25^a^	73.88 ± 0.69^a^	74.12 ± 1.33^a^	0.042	0.272	0.270
**ADFI (g)**									
1–14 d	69.81 ± 1.30	70.17 ± 1.42	67.61 ± 1.04	69.40 ± 1.49	67.69 ± 0.91	68.40 ± 0.88	0.530	0.192	0.427
15–35 d	173.60 ± 4.07	178.32 ± 2.48	174.39 ± 3.21	174.23 ± 3.47	175.54 ± 1.79	176.85 ± 3.33	0.892	0.661	0.905
1–35 d	133.93 ± 1.69	136.32 ± 1.66	132.96 ± 2.23	133.56 ± 2.64	133.67 ± 1.23	134.70 ± 2.00	0.866	0.766	0.840
**F/G**									
1–14 d	1.55 ± 0.02	1.56 ± 0.02	1.55 ± 0.01	1.54 ± 0.01	1.53 ± 0.02	1.57 ± 0.01	0.651	1.000	0.626
15–35 d	2.02 ± 0.04	1.91 ± 0.01	1.90 ± 0.01	1.87 ± 0.04	1.91 ± 0.01	1.90 ± 0.02	0.171	0.164	0.056
1–35 d	1.90 ± 0.03^a^	1.82 ± 0.01^b^	1.81 ± 0.01^b^	1.78 ± 0.03^b^	1.81 ± 0.01^b^	1.82 ± 0.01^b^	0.041	0.199	0.024

*Results are presented as mean ± SEM (n = 6), values in the same row with different superscript letters are significantly different (P < 0.05) as determined by ANOVA and Duncan's test*.

*BW, Body weight; ADG, Average daily gain; ADFI, Average daily feed intake; F/G, Feed to gain ratio*.

### Effects of Zn-Met on the Intestinal Antioxidant Capacity in Meat Ducks

The effect of Zn-Met on intestinal antioxidant enzyme activity of meat ducks are shown in Table 5. Dietary Zn-Met markedly increased the activity of SOD, CAT and GSH in the jejunum (*P* < 0.05), while the content of MDA in the jejunum was significantly reduced (*P* < 0.05). There was no significant difference in the activities of T-AOC and GSH-Px in the jejunum between all treatments (*P* > 0.05). The activity of GSH-Px in the ileum was significantly increased (*P* < 0.05), and the content of MDA was decreased in the Zn-Met dietary groups.

**Table 5:** Effect of dietary Zn-Met on the antioxidant status in the intestine of ducks.

**Items**	**Dietary Zn levels (mg/kg)**						***P*** **-value**		
	**0**	**30**	**60**	**90**	**120**	**150**	**AVONA**	**Linear**	**Quadratic**
**Jejunum**									
T-AOC (U/mg prot)	18.92 ± 0.91	20.85 ± 0.88	21.54 ± 1.23	24.03 ± 0.89	21.82 ± 1.61	21.75 ± 0.79	0.069	0.178	0.085
SOD (U/mg prot)	50.72 ± 0.93^b^	51.02 ± 4.82^b^	58.86 ± 4.74^ab^	67.78 ± 3.28^a^	62.15 ± 3.91^ab^	57.38 ± 2.74^ab^	0.019	0.194	0.144
CAT (U/mg prot)	24.29 ± 1.96^c^	23.47 ± 1.15^c^	32.96 ± 3.21^a^	32.50 ± 1.92^ab^	26.23 ± 2.73^bc^	23.55 ± 1.51^c^	0.006	0.925	0.217
GSH (mg/g prot)	48.58 ± 5.28^c^	53.79 ± 5.38^bc^	77.47 ± 2.61^a^	63.95 ± 7.17^abc^	71.58 ± 7.40^ab^	72.84 ± 8.36^ab^	0.017	0.085	0.170
GSH-Px (U/mg prot)	25.75 ± 1.31	30.49 ± 3.08	30.79 ± 2.83	37.74 ± 2.61	31.43 ± 3.30	32.26 ± 1.77	0.079	0.220	0.176
MDA (nmol/mg prot)	5.63 ± 0.84^a^	3.28 ± 0.52^b^	3.53 ± 0.57^b^	3.30 ± 0.54^b^	3.67 ± 0.56^b^	2.55 ± 0.36^b^	0.021	0.090	0.207
**Ileum**									
T-AOC (U/mg prot)	14.73 ± 1.06	13.22 ± 1.09	11.28 ± 0.40	14.04 ± 1.86	12.61 ± 1.50	12.19 ± 0.92	0.411	0.312	0.542
SOD (U/mg prot)	44.94 ± 2.54	57.76 ± 3.03	50.00 ± 3.61	55.09 ± 5.99	57.53 ± 4.03	60.39 ± 6.11	0.125	0.081	0.273
CAT (U/mg prot)	23.51 ± 1.64	24.62 ± 1.08	22.36 ± 1.41	24.19 ± 2.39	24.97 ± 2.49	23.32 ± 2.42	0.945	0.839	0.972
GSH (mg/g prot)	54.29 ± 3.00	63.94 ± 4.09	54.40 ± 4.98	60.87 ± 2.16	59.83 ± 3.20	66.36 ± 3.47	0.129	0.215	0.483
GSH-Px (U/mg prot)	24.38 ± 1.26^b^	32.60 ± 1.83^a^	27.56 ± 2.23^ab^	33.70 ± 2.48^a^	33.39 ± 2.96^a^	30.05 ± 2.45^ab^	0.040	0.279	0.310
MDA (nmol/mg prot)	4.59 ± 0.29^a^	3.10 ± 0.41^bc^	3.16 ± 0.32^bc^	3.88 ± 0.44^ab^	3.68 ± 0.37^ab^	2.13 ± 0.40^c^	0.002	0.180	0.461

*Results are presented as mean ± SEM (n = 6), values in the same row with different superscript letters are significantly different (P <0.05) as determined by ANOVA and Duncan's test*.

*T-AOC, total antioxidant capacity; SOD, superoxide dismutase; CAT, catalase; GSH, glutathione; GSH-Px, glutathione peroxidase; MDA, malondialdehyd*.

**Figure 1:**
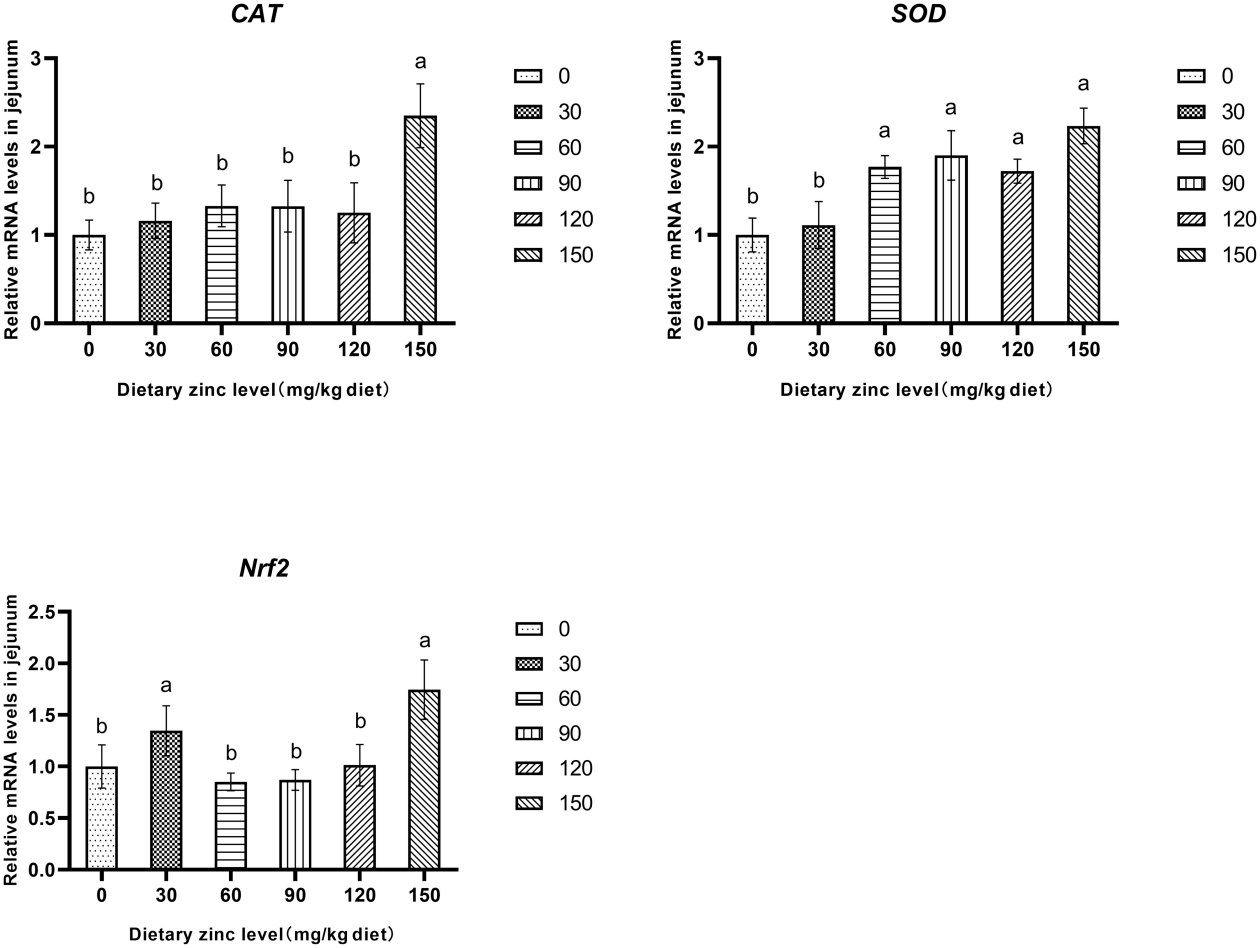
Effects of Zn-Met on the gene expression of antioxidase activity in jejunum of ducks. CAT, catalase; SOD, superoxide dismutase; Nrf2, nuclear factor erythroid 2-related factor 2.

Further research found that the 150 mg/kg Zn significantly improved the mRNA expression of *CAT* in the jejunum (*P* < 0.05). Meanwhile, the groups provided with 60, 90, 120 and 150 mg/kg of Zn had a significant increase of the *SOD* mRNA expression in the jejunum (*P* < 0.05). It was also observed that supplementing a certain level of Zn improved the mRNA expression of *Nrf2* in the jejunum (*P* < 0.05) (Figure 1).

### Effects of Zn-Met on Intestinal Permeability and Physical Barrier Function in Meat Ducks

**Figure 2:**
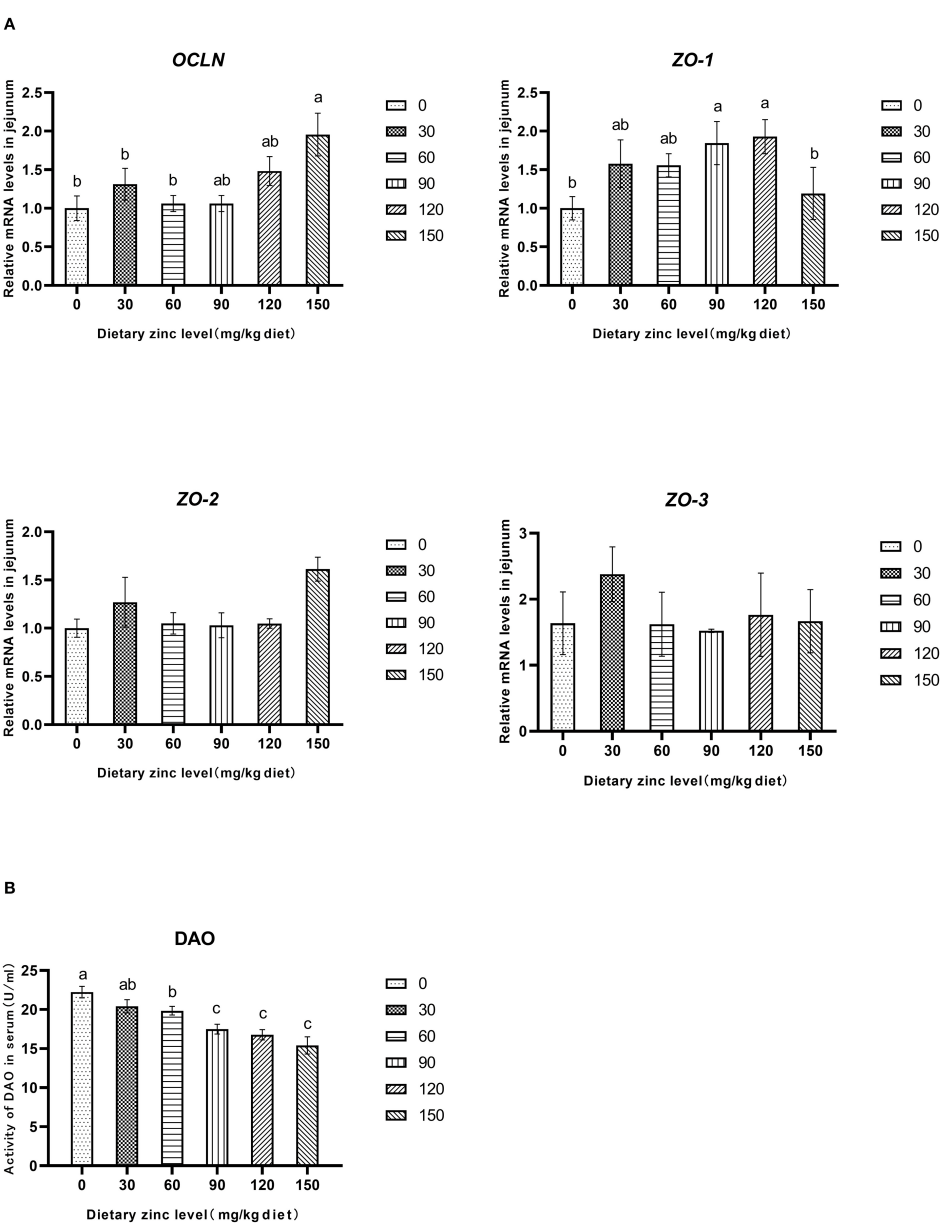
Effects of Zn-Met on gene expression and permeability related to physical barrier of jejunum in meat duck. OCLN, occludin; ZO-1, zonula occludens-1; ZO-2, zonula occludens-2; ZO-3, zonula occludens-3; DAO, diamine oxidase.

The effects of Zn-Met on intestinal permeability and tight junction protein gene expression of meat ducks are shown in Figure 2. Compared with the basal diet group, adding different levels of Zn increased the mRNA expression of *OCLN* and *ZO-1* in the jejunum. When the addition amount reached 150 mg/kg, the gene level of *OCLN* significantly improved (*P* < 0.05). Different levels of Zn had no significant effect on the mRNA expression of *ZO-2* and *ZO-3* in the jejunum (*P* > 0.05) (Figure 2A). Supplementation with 60, 90, 120 and 150 mg/kg of Zn significantly reduced the DAO activity in serum (*P* < 0.05), and the DAO activity in serum decreased linearly with the increase of Zn level (*P* < 0.05) (Figure 2B).

### Effects of Zn-Met on Intestinal Chemical Barrier Function in Meat Ducks

The effect of dietary Zn-Met on intestinal mucin content of ducks are shown in Table 6. As the level of Zn-Met increased, the content of MUC2 in the jejunum and ileum increased significantly (*P* < 0.05). When the supplemental levels were 90, 120 and 150 mg/kg, no significant difference among the treatment groups was observed (*P* > 0.05).

**Table 6:** Effect of dietary Zn-Met on intestinal mucin content of ducks.

**Items**	**Dietary Zn levels (mg/kg)**						***P*** **-value**		
	**0**	**30**	**60**	**90**	**120**	**150**	**AVONA**	**Linear**	**Quadratic**
**Jejunum**									
MUC2 (ng/mL)	20.37 ± 1.51^c^	25.30 ± 1.68^b^	25.40 ± 1.16^b^	27.60 ± 1.18^ab^	30.50 ± 1.40^a^	29.76 ± 1.34^a^	<0.0001	0.006	0.018
**Ileum**									
MUC2 (ng/mL)	14.88 ± 2.03^c^	20.25 ± 1.31^b^	24.14 ± 1.82^ab^	25.52 ± 1.30^a^	27.17 ± 1.48^a^	25.42 ± 1.46^a^	<0.0001	0.022	0.001

*Results are presented as mean ± SEM (n = 6), values in the same row with different superscript letters are significantly different (P <0.05) as determined by ANOVA and Duncan's test*.

### Effect of Dietary Zn-Met on Intestinal Immune Barrier Function in Meat Ducks

Table 7 shows the effect of dietary Zn-Met on intestinal immunoglobulin content in meat ducks. As the level of Zn-Met increased, the content of sIgA, IgA and IgG in the jejunum of meat ducks increased significantly (*P* < 0.05). Compared with the basal diet group, the addition of 60, 90, 120 and 150 mg/kg Zn-Met significantly increased the IgM content in the jejunum (*P* < 0.05), while the addition of 30 mg/kg Zn had no significant effect on the IgM (*P* > 0.05). The content of sIgA and IgG in the ileum showed linear and quadratic changes with the increase of Zn level (*P* < 0.05). Compared with the basal diet group, more than 60 mg/kg Zn significantly increased the content of IgA and IgM in the ileum (*P* < 0.05).

**Table 7:** Effect of dietary Zn-Met on intestinal immunoglobulin content in ducks.

**Items**	**Dietary Zn levels (mg/kg)**						***P*** **-value**		
	**0**	**30**	**60**	**90**	**120**	**150**	**AVONA**	**Linear**	**Quadratic**
**Jejunum**									
sIgA (ng/mL)	31.11 ± 2.28^e^	34.53 ± 1.31^de^	38.28 ± 2.64^cd^	52.01 ± 2.50^a^	47.26 ± 2.29^ab^	44.50 ± 2.50^bc^	<0.0001	0.059	0.097
IgA (ug/mL)	236.72 ± 8.76^d^	272.35 ± 9.99^c^	290.50 ± 14.68^bc^	334.92 ± 11.71^a^	316.70 ± 12.40^ab^	309.58 ± 12.24^ab^	<0.0001	0.044	0.020
IgG (ug/mL)	1201.27 ± 86.00^d^	1501.70 ± 61.91^c^	1722.77 ± 62.90^bc^	2005.59 ± 101.84^a^	1946.71 ± 126.68^ab^	1573.34 ± 88.17^c^	<0.0001	0.189	0.023
IgM (ug/mL)	376.84 ± 19.12^b^	345.92 ± 14.07^b^	451.18 ± 11.39^a^	453.14 ± 19.41^a^	469.09 ± 17.21^a^	434.95 ± 23.02^a^	<0.0001	0.106	0.200
**Ileum**									
sIgA (ng/mL)	31.10 ± 1.37^b^	34.18 ± 2.31^b^	37.30 ± 2.38^b^	48.56 ± 1.99^a^	45.60 ± 1.95^a^	44.00 ± 2.84^a^	<0.0001	0.033	0.070
IgA (ug/mL)	251.06 ± 9.33^c^	276.28 ± 10.06^bc^	286.26 ± 13.24^b^	340.84 ± 11.96^a^	357.40 ± 12.13^a^	300.99 ± 11.23^b^	<0.0001	0.101	0.129
IgG (ug/mL)	1123.18 ± 111.79^b^	1284.79 ± 93.32^b^	1634.23 ± 67.33^a^	1859.03 ± 87.79^a^	1834.67 ± 98.46^a^	1674.20 ± 98.47^a^	<0.0001	0.043	0.016
IgM (ug/mL)	336.46 ± 16.96^c^	377.43 ± 11.52^bc^	397.25 ± 16.17^b^	479.00 ± 32.17^a^	471.66 ± 14.99^a^	406.73 ± 12.06^b^	0.001	0.126	0.089

*Results are presented as mean ± SEM (n = 6), values in the same row with different superscript letters are significantly different (P < 0.05) as determined by ANOVA and Duncan's test*.

*SlgA, Secretory immunoglobulin A; IgA, Immunoglobulin A; IgG, Immunoglobulin G; IgM, Immunoglobulin M*.

### Effects of Zn-Met on Intestinal Microflora of Meat Ducks

#### Effective Sequence of Cecal Microorganisms of Meat Ducks

In this sequencing, the six treatment groups adding 0, 30, 60, 90, 120, and 150 mg/kg Zn-Met produced 502405, 498146, 456926, 508847, 588034, and 505485 original sequences, and the effective sequences obtained through quality control were 439509, 425070, 368555, 420248, 500787, and 441886, with an average effective rate of 87.48, 85.33, 80.66, 82.59, 85.16, and 87.42%, respectively, indicating the high quality of sequencing data.

**Figure 3:**
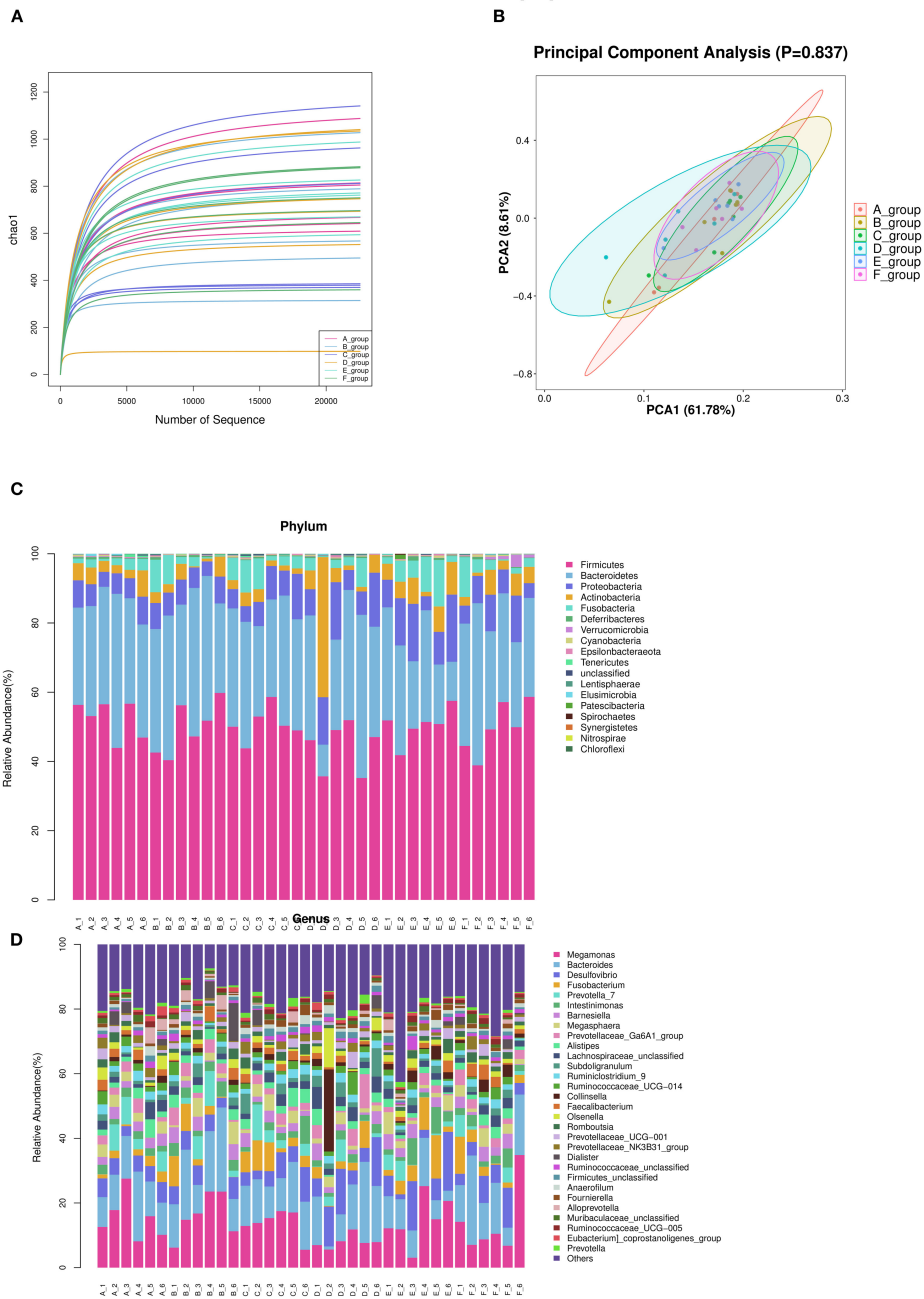
Summary of bacterial taxa in duck cecal digesta observed by concentration of Zn-Met in the diet. **(A)** The dilution curve was constructed according to the number of randomly selected sequences and the number of observed features. **(B)** PCA among three groups based on Weighted UniFrac distances. Each point represented a sample. **(C)** Depicts phylum level classifications for observed features. **(D)** Depicts genus level classifications for observed features.

According to the number of randomly selected sequences and the number of observed features values, the rarefaction curve was constructed. To a certain extent, the curve can directly reflect whether the amount of sequencing data is reasonable or not, and indirectly reflect the species richness of samples. The curve of each treatment tends to be flat, reaching the platform stage, indicating that the sequencing depth has basically covered all species, and the sequencing quantity tends to be saturated (Figure 3A).

#### Alpha Diversity Analysis

The abundance and evenness of microbial communities were mainly reflected by Chao1, Shannon and Simpson indices. As shown in Table 8, the indexes of Chao1 (*P* = 0.97), Shannon (*P* = 0.95) and Simpson (*P* = 0.91) among the treatment groups had no significant difference (*P* > 0.05), indicating that different levels of Zn-Met had no significant effect on the richness and evenness of cecal microbial community of meat ducks.

**Table 8:** Effect of dietary Zn-Met on the diversity of cecal digesta microbiota in ducks.

**Items**	**Dietary Zn levels (mg/kg)**						***P-*value**
	**0**	**30**	**60**	**90**	**120**	**150**	
Chao1	768.63 ± 73.46	665.64 ± 105.91	668.95 ± 134.92	692.66 ± 141.91	762.17 ± 52.73	709.89 ± 80.45	0.97
Shannon	7.17 ± 0.22	6.84 ± 0.26	7.12 ± 0.16	7.04 ± 0.35	7.09 ± 0.21	7.11 ± 0.22	0.95
Simpson	0.97 ± 0.01	0.97 ± 0.01	0.98 ± 0.01	0.98 ± 0.004	0.97 ± 0.01	0.97 ± 0.01	0.91

*Results are presented as mean ± SEM (n = 6), values in the same row with different superscript letters are significantly different (P <0.05) as determined by ANOVA and Duncan's test*.

#### β Diversity Analysis

The principal component analysis diagrams of each treatment group were relatively concentrated, most of them were overlapped, and there was no significant difference, indicating that there was no significant difference in cecal microflora of meat ducks with different levels of Zn-Met under the experimental conditions (*P* > 0.05) (Figure 3B).

#### Structural Composition of Cecal Microflora

##### Phylum Level

According to the species abundance table and annotation table, 30 species with the highest abundance were selected for classification. As shown in Figure 3C and Table 9, at the phylum level, the top 10 flora with relative abundance in each treatment group were *Firmicutes, Bacteroidetes, Proteobacteria, Actinobacteria, Fusobacteria, Deferribcteres, Verrucomicrobia* and *Cyanobacteria Epsilonbacteraeota* and *Tenericutes*. The relative abundance of *Firmicutes, Bacteroidetes, Proteobacteria* and *Actinobacteria* accounted for more than 90% of the total cecal microorganisms in meat ducks. Addition of Zn-Met has a trend to increase the relative abundance of Proteobacteria in the cecum of meat ducks. As shown in Table 9, when the addition amount reached 150 mg/kg, Zn-Met significantly increased the relative abundance of *Verrucomicrobia* in the cecum (*P* < 0.05).

**Table 9:** The relative abundance at the phylum level of cecal microbe.

**Items**	**Dietary zinc level(mg/kg)**						**SEM**	***P*-value**
	**0**	**30**	**60**	**90**	**120**	**150**		
*Firmicutes*	52.26	49.67	50.79	44.20	50.48	49.73	1.07	0.366
*Bacteroidetes*	33.56	36.18	32.47	31.32	24.09	32.50	3.22	0.247
*Proteobacteria*	6.39	6.55	7.85	11.00	11.87	8.06	0.67	0.067
*Actinobacteria*	4.22	2.83	2.47	9.62	5.84	4.04	1.09	0.450
*Fusobacteria*	2.11	3.82	5.33	2.82	6.32	3.78	0.60	0.350
*Deferribacteres*	0.44	0.38	0.26	0.28	0.43	0.26	0.04	0.651
*Verrucomicrobia*	0.15^b^	0.08^b^	0.04^b^	0.06^b^	0.08^b^	1.03^a^	0.10	0.018
*Cyanobacteria*	0.15	0.11	0.17	0.13	0.24	0.25	0.03	0.700
*Epsilonbacteraeota*	0.14	0.11	0.19	0.21	0.23	0.11	0.02	0.483
*Tenericutes*	0.25	0.10	0.17	0.06	0.04	0.10	0.03	0.223

*Results are presented as mean ± SEM (n = 6), values in the same row with different superscript letters are significantly different (P < 0.05) as determined by ANOVA and Duncan's test*.

##### Genus Level

It can be seen from Figure 3D that at the genus level, the top 10 flora with relative abundance in each treatment group were *Megamonas, Bacteroides, Desulfovibrio, Fusobacterium* and *Prevotella_ 7, Intestinimonas, Barnesiella, Megasphaera, Prevotellaceae_ Ga6A1_ Group*, and *Alistipes*. There was no significant difference in the relative abundance among the top 10 treatment groups (*P* > 0.05). However, the relative abundance of *Akkermansia* in cecum was significantly increased by adding 150 mg/kg Zn (*P* < 0.05).

#### Estimation of the Dietary Zn Requirements

The suitable addition amount of Zn determined according to different effect indexes are shown in Table 10. Under the condition of this experiment, through quadratic regression analysis and the establishment of univariate quadratic equation, taking F/G, jejunal IgA, jejunal IgG and ileal IgG as the effect index, the supplemental amount of Zn in the basic diet of meat ducks were 83.33, 113.21, 93.70, and 110.10 mg/kg, respectively.

**Table 10:** The optimal dietary Zn-Met supplementation based on different indices for ducks.

**Items**	**Regression equation^a^**	**R^**2**^**	***P*-value**	**Optimal dietary Zn-Met levels (mg/kg diet)**
F/G (g:g)	y = 1.210E – 5x^2^ – 0.002x + 1.892	0.916	<0.05	83.33
IgA of content in jejunum(ng/mL)	y = −0.007x^2^ + 1.585x + 233.381	0.925	<0.05	113.21
IgG of content in jejunum (ng/mL)	y = −0.089x^2^ + 16.672x + 1142.930	0.920	<0.05	93.70
IgG of content in ileum (ng/mL)	y = −0.062x^2^ + 13.652x + 1052.811	0.936	<0.05	110.10

a *y Is the Dependent Variable and x Are the Dietary Zn Supplemental Levels (mg/kg)*.

*Results are presented as mean ± SEM (n = 6), values in the same row with different superscript letters are significantly different (P <0.05) as determined by ANOVA and Duncan's test*.

## Discussion

### Effects of Zn-Met on Growth Performance of Meat Ducks

Studies have shown that a certain level of Zn-Met can improve the growth performance of broilers (31). Zn is a component of more than 300 enzymes. These enzymes are involved in the metabolism of protein, energy, carbohydrate and nucleic acid (32). Therefore, the possible reason why Zn can improve the growth performance of animals is that Zn participates in the synthesis of nucleic acid and protein, energy metabolism, redox and other biochemical metabolic processes in the body, thus affecting the material metabolism of the body and the growth and development of animals. The results showed that the appropriate level of Zn-Met significantly increased the final body weight and average daily gain of meat ducks, significantly reduced the F/G, and improved the growth performance of meat ducks.

### Effects of Zn-Met on Intestinal Antioxidant Capacity of Meat Ducks

Oxidative stress is a key factor in the destruction of intestinal barrier function (33). Zn is an important component of antioxidant enzymes in cells, which plays an important role in scavenging superoxide free radicals and protecting cells from oxidative stress (34). SOD can convert ROS into hydrogen peroxide, which is degraded into water and oxygen by CAT and GSH-Px (35), GSH is a scavenger *in vivo*, which can scavenge superoxide and hydroxyl radicals (36). The increase of MDA concentration is an important indicator of lipid peroxidation (37), reducing the content of MDA is helpful to improve the body's antioxidant capacity. The results showed that Zn-Met could increase the activities of SOD, CAT and GSH, significantly reduce the content of MDA, and improve the antioxidant capacity of intestine. *Nrf2* is a key transcription factor that regulates the expression of antioxidant enzymes (22). The results showed that ZnSO_4_ could induce the expression of antioxidant enzymes in breast muscle of Peking Duck by increasing *Nrf2* gene expression (38). It was found that Zn-Met could significantly increase the gene expression of *Nrf2, SOD* and *CAT* in the intestine. In conclusion, Zn-Met may affect *Nrf2* signaling pathway and improve the antioxidant capacity.

### Effects of Zn-Met on Intestinal Barrier Function of Meat Ducks

The integrity of intestinal barrier is closely related to intestinal health. The destruction of intestinal physical barrier will lead to the release of a large number of DAO and active substances, and the content of DAO in serum is an effective biomarker to measure the integrity of small intestine and intestinal barrier function (39). In this experiment, adding different levels of Zn-Met decreased the activity of DAO in serum of meat ducks, which indicated that Zn-Met could reduce the intestinal permeability and improve the intestinal barrier function of meat ducks. In addition, tight junction protein is an important component of intestinal physical barrier, and Zn has a certain repair effect on intestinal physical barrier (40). The results showed that the addition of Zn could significantly increase the mRNA expression of *OCLN* and *CLDN-1*, and alleviate the intestinal mucosal barrier function damage caused by *Salmonella typhimurium* infection in broilers (41). In this study, Zn-Met significantly increased the mRNA expression of *OCLN* and *ZO-1* in the jejunum of ducks. In conclusion, Zn-Met can enhance the physical barrier function of meat ducks by reducing the intestinal permeability and increasing the gene expression of tight junction proteins.

MUC2 is the main component of intestinal mucus layer. MUC2 is distributed in small intestine and large intestine, forming mucus skeleton, which is the main component of intestinal chemical barrier (42). The mucous layer covering the intestinal epithelial cells can prevent the invasion of toxic substances, digestive enzymes and bacteria (43). Studies on pigs and chickens showed that dietary Zn supplementation could increase the content and gene expression of *MUC2* in the intestine (44, 45). The results showed that the contents of MUC2 in jejunum and ileum of meat ducks increased significantly with the increase of Zn-Met level, which enhanced the chemical barrier of meat ducks.

Zn homeostasis is essential for maintaining normal immune system function. Both Zn deficiency and excess lead to severe disorder of the number and activity of immune cells, leading to the occurrence of inflammatory diseases (46). Immunoglobulin is a protein which is composed of two identical heavy chains and light chains connected by disulfide bonds. It can be divided into five subtypes, including IgA, IgG, IgM, IgE and IgD (47), it is an important part of the immune system. sIgA is the dominant antibody in intestinal mucosal secretions, which can prevent the adhesion and penetration of pathogens into the intestinal barrier (48). Studies have shown that Zn deficiency can reduce the content of immunoglobulin in animal intestines (49), and Zn addition can improve the content and gene expression of immunoglobulin in pigs and poultry intestines (50, 51). The results showed that Zn-Met could significantly increase the contents of SlgA, IgA, IgM and IgG in the jejunum and ileum of meat ducks, and enhance the immune function of meat ducks.

Intestinal microflora is a dynamic and relatively stable system. Studies have shown that zinc deficiency can cause significant taxonomic changes in cecal microflora of broilers, and reduce the richness and diversity of the whole microflora (26). A large number of studies have shown that zinc can affect the composition of intestinal microflora and promote intestinal health of animals (52). The interaction between intestinal microflora and minerals will affect the host's health (52). The intestinal tract is an important organ for Zn absorption. Zn deficiency can change the composition and function of intestinal flora in broilers, mainly in that Zn deficiency can cause significant taxonomic changes in the cecum of broilers, reduce species richness and diversity (26), and then lead to intestinal flora disorder and damage the intestinal health of animals. Studies have found that *Akkermansia* is a major member of *Verrucomicrobia*, which can use mucin as the only source of carbon and nitrogen, and stimulate the expression of mucin, it is a potential probiotic (53). Yu et al. (27) found that high dose of Zn oxide can significantly increase the phase of *Verrucomicrobia* in ileal chyme of weaned piglets for abundance, it can regulate the intestinal microflora of piglets. In addition, this study showed that adding 150 mg/kg Zn-Met could significantly increase the relative abundance of *Verrucomicrobia* and *Akkermansia* at the level of mesocaecum, suggesting that Zn-Met could promote the secretion of intestinal mucin by increasing the relative abundance of *Akkermansia*.

## Conclusion

In conclusion, the optimal supplemental Zn should be 83.33 mg/kg, according to the optimum F/G during 1–35 d for meat ducks. Appropriate levels of Zn not only improved the growth performance of meat ducks, but also increased the intestinal antioxidant capacity, physical barrier, chemical barrier and immune function. An improved cecum microbial profile was also observed under the appropriate Zn concentrations. In addition, Zn-Met improves the antioxidant capacity of the jejunum by activating the *Nrf2* signaling pathway. These findings provide a valuable reference for research on the mechanisms by which Zn-Met improves the intestinal health and growth of meat ducks.

## Data Availability Statement

The datasets presented in this study can be found in online repositories. The names of the repository/repositories and accession number(s) can be found below: https://www.ncbi.nlm.nih.gov/, XM_013109403.1; XM_013104936.1; XM_013093747.1; XM_005019888.2; XM_005013555.2; XM_013097859.1; KU048802.1; EF667345.1.

## Ethics Statement

The animal study was reviewed and approved by Animal Health and Care Committee of Sichuan Agricultural University (No. 20181203). Written informed consent was obtained from the owners for the participation of their animals in this study.

## Author Contributions

HT: conceptualization, methodology, and writing-original draft preparation. YC and ZZ: software, data curation, and revising the manuscript. TY, FW, and HZ: visualization and investigation. BW, GL, XC, GT, and JC: supervision, software, and validation. GJ: writing-reviewing and editing. All authors contributed to the article and approved the submitted version.

## Funding

This work was supported by Sichuan Longda Animal Husbandry Science and Technology Co., Ltd. (No. 009H2200).

## Conflict of Interest

BW was employed by Chelota Group. The remaining authors declare that the research was conducted in the absence of any commercial or financial relationships that could be construed as a potential conflict of interest.

## Publisher's Note

All claims expressed in this article are solely those of the authors and do not necessarily represent those of their affiliated organizations, or those of the publisher, the editors and the reviewers. Any product that may be evaluated in this article, or claim that may be made by its manufacturer, is not guaranteed or endorsed by the publisher.
